# Ion Release, Microhardness and Enamel Demineralization Resistance of New Bioactive Restorative Materials 

**DOI:** 10.4317/jced.62357

**Published:** 2025-06-01

**Authors:** Tayseer Maaly, Fawzy A. Darweesh, Mohamed Samir Elnawawy

**Affiliations:** 1 Dental Biomaterials, Faculty of Dentistry, Zagazig University, Egypt; 2 Oral Biology Department, Faculty of Dentistry, Zagazig University, Egypt; 3 Clinical Dentistry Department, General Dentistry Program, Batterjee Medical College, Jeddah21442, Sausi Arabia. Conservative Department, Faculty of Dentistry, Mansoura University, Egypt

## Abstract

**Background:**

The objective of the study was to assess fluoride and calcium release from new bioactive materials and to correlate the results with surface hardness and enamel demineralization resistance.

**Material and Methods:**

Three ion releasing restorative materials were considered: Surefil one (SO), Equia forte fil HT (EF), and Activa bioactive resorative (AB). Baseline microhardness (MH) of the restorative materials was recorded. The amount of released fluoride and calcium ions and microhardness of the materials were estimated at different intervals of 7,14 and 21 days storage in distallid water. Ion release values were recorded using Ionchromatography. Finally, enamel demineralization resistance was evaluated using a microhardness tester. Enamel surface morphology, calcium and phosphorous wt. % were evaluated utilizing the scanning electron microscopy with energy dispersive X-ray spectroscopy (SEM\EDX).

**Results:**

SO released more fluoride and lower calcium ions than the other groups (*p*< 0.05). EF recorded more fluoride and calcium ion release compared to AB. The highest MH values at all intervals were for SO followed by EF, both materials exhibited significant MH increase upon storage. AB exhibited the lowest MH which decreased upon storage. In the acid resistance test, EF showed effective resistance to demineralization followed by AB.

**Conclusions:**

EF is an effective restorative material when applied in cariogenic media with adequate surface hardness qualities upon storage.

** Key words:**Bioactive material, Ion release, Enamel demineralization resistance, Microhardness.

## Introduction

Secondary caries is a common cause of failure of permanent dental restorations. The ability of restorative materials to prevent demineralization of dental hard tissues by release of ions and acid neutralization is a practical way to prolong the service life of restorations (1,2). Currently, the most prevalent direct restorative material for treating carious lesions that cannot be stopped or remineralized is resin composite (3). However, these resins do not release fluoride so secondary caries inhibition is a serious concern. Additionally, polymerization shrinkage might compromise restoration margins. As a result, manufacturers have created contemporary dental restorative materials with bioactive ingredients and resin-based materials that include glass filler. These materials have shown antimicrobial activity against oral bacteria, which helps to reduce demineralization of nearby teeth (4). Moreover, these unique restoratives keep the value of being bulk filled, therefore, excludes the difficulties and problems related to incremental filling, as contamination, time-consuming filling, and interlayer voids (5).

In order to combine the crosslinking capability of essential monomers of composites with the self-adhesive qualities of conventional polyacids compound of glass ionomer cements, Surefil One, a composite hybrid with self adhesive qualities, was developed and patented (6). Equia Forte HT is a glass ionomer cement which has high viscosity and releasing F and Ca , that cures itself without the need for resin. Various sizes of glass particles such as highly reactive tiny particles mixed to the conventional filler, have been used to modify glass hybrid materials based on GIC technology (7). ACTIVA-Bioactive Restorative is a bioactive dental material composed of an ionic resin matrix in addition to bioactive fillers which imitate the chemical and physical characteristics of natural teeth (8,9). It was claimed that the formation of microcracking and a decrease in mechanical characteristics could result from the ions release from bioactive materials (10). Over time, ion leaching from the material may affect several qualities, hence jeopardizing lifetime (11). Degradation affects a number of RBC characteristics, such as flexural strength, color stability, dimensional stability, wear resistance, and surface hardness (12). Because the ions discharged may cause voids inside the tooth material, questions regarding their mechanical and physical performances were raised (12). For this reason, surface hardness should be taken into account in addition to a restorative material’s antibacterial qualities. A restorative material’s resistance to wear and scratches is increased when it has the perfect surface microhardness (13).

Although calcium and fluoride can be released by Surefil One, Equia Forte HT filling, and ACTIVA, it is unknown if these materials can prevent enamel demineralization at restorative margins. Therefore, to determine the impact of these novel bioactive restorative materials in preventing formation of secondary caries, an assessment of the demineralization process is necessary. So, this study was conducted to assess fluoride and calcium ion release from bioactive restorative materials, the ability of these materials to resist enamel demineralization and evaluation of microhardness of the materials after water storage. The null hypothesis was that all materials would record the same ions releasing potential and the released ions had no effects on the enamel demineralization resistance or microhardeness of the tested materials.

## Material and Methods

Materials utilized in our study are listed in  Table 1. The study was conducted at Dental Biomaterials Deparment, Faculty of Dentistry, Zagazig University, Egypt. The study’s methods were authorized by the Institutional Review Board of Zagazig University’s Faculty of Medicine under ethical number 11221-24-10-2023.

- Sample Size Calculation:

Based on a recent study, the G*Power 3 sample size calculator indicated that the accepTable sample size was 6 specimens per group at error prop (α) = 0.01 and power (1-β) = 0.99 of the study. (14) To make up for missing data and pre-test failures, it was raised to 10 specimens per group. Additionally, using the G*Power 3 sample size calculator, the sample size of the extracted sound permanent teeth was determined to be 20 specimens for each group at error prop (α) = 0.05 and power (1-β) = 0.90 of the study, based on the enamel’s microhardness values following demineralization (2).

- Preparation of test specimens

A standardized split Teflon mold was utilized to create cylindrical specimens ( 10 mm in diameter and 3 mm in height), n = (90) for the fluoride release and surface hardness tests (15). To stop material adherence, coating of petroleum jelly was performed to the mold’s lateral walls. All of the tested components were in encapsulated form and were combined using a capsule mixer (GC Corporation Tokyo, Japan) in line with the manufacturer’s instructions. To stop the material from sticking to the glass plate, Mylar strips were positioned between the Teflon mold and the glass plate after the materials had been dispensed in the mold and slightly overflowed. In order to prevent air bubbles and attain a flat surface, glass plates were held tightly during the setting process. The light cure equipment (Elipar S10, 3M ESPE; USA, wavelength 455 nm ± 10 nm, light intensity 1200 mW/cm²) was chosen to cure the materials for 40 seconds.

Specimens were separated into 3 groups (n = 30) relative to the restorative materials. Three subgroups were then formed from each group based on the time of storage: 7, 14, and 21 days. Baseline microhardness (MH) readings were taken by a Vickers Micro-hardness Instrument (FM-700, Kawasaki, Kanagawa, Japan) with a load of 300 g at 23±1 °C for 15 s after drying at 37±1 ◦C for one hour. Every specimen was dipped in a bottle filled with five milliliters of distilled water, and the solution remained constant throughout the test (2). At 7, 14, and 21 days, the cumulative emission of calcium ions and fluoride was measured (mg/L). Ion release was evaluated using an ICS-5000DC ion chromatography model.

The microhardness of the specimens utilized in the ion assess at the three time intervals was measured. Each specimen’s surface hardness was measured after 7, 14, and 21 days. Specimens were taken out of the vials at each time interval and given a minute to dry before testing. On each specimen, three indentations were created at each time period. This equation was used to determine Vicker hardness number (VHN): H = 1854.4 P d2, where d is the diagonal length in µm, P represents the load in grams, and H is the Vickers hardness in kg/mm2.

- Evaluation of enamel demineralization resistance

Specimens prepearation: This study included upper and lower molars that had recently been extracted because of periodontal disease and had roughly comparable size. After being checked for cavities and cracks using a stereomicroscope, teeth were washed using an ultrasonic scaler to get rid of calculus and soft tissue buildup. They were then kept in a 0.5% Chloramine T solution until they could be tested experimentally. Crown was separated from the root with a low-speed water-cooled diamond disc. Crown were inserted in self-polymerized acrylic resin in a plastic mold to simplify processing. Abrasive paper with grits of 400, 800, and 1200 was used to polish the crowns. To standardize the optical surface properties for the microhardness test following polishing, cavity was created in the middle of the enamel. Class V preparation was done on each tooth’s buccal surfaces using carbide bur (#330), with a high-speed hand piece. Each test material was then inserted into the cavity. Materials were arranged following the manufacturer’s guidelines. Sof-LexTM discs (3M ESPE, St. Paul, MN, USA) were used to polish every restoration. Acid-protective varnish was applied to the teeth, leaving at least 1 mm of exposed space next to the restoration margins. After that, the teeth were submerged in the demineralization solution for 21 days. The solution composition was 50 mM acetic acid, 2.2 mM CaCl2, and 2.2 mM NH2PO4. Next, 1 M KOH was used to bring the demineralization solution’s pH down to 4.4 (2).

Prior to and following immersion in demineralization solution , the enamel surface’s microhardness was tested. A Vickers hardness tester with a 100 g load and a 10-second dwell period was used to record the measurements. To quantitatively evaluate each specimen’s restoration/tooth interface, the EDS spectrometer was connected to a scanning electron microscope. The SEM-EDX (TESCAN VEGA 3, Czech Republic) was used to measure the calcium and phosphorus weight percentages on the demineralized enamel surface at 20 kV voltage and a magnification of 500×. The EDX detector produced a histogram plot to show the weight percentages of calcium and phosphate. The enamel surfaces of the specimens were examined using SEM. Before analysis, the specimens were coated with gold (Au) using a Quorum Methods Ltd. sputter coater (Q150t, England) and affixed to aluminum stubs. Microphotographs were taken at a 100x magnification for every group.

- Statistical analysis:

Data were analyzed by Version 2.0 of IBM SPSS Statistics for Windows. Data were analyzed by One-Way ANOVA test at a significance level of 0.05. Several comparisons were performed via Tukey’s post-hoc analysis. Ion release, microhardness, and enamel demineralization resistance were all correlated using Pearson’s correlation coefficient. Significant differences detected as *P*-values under 0.05.

## Results

1. Fluoride and calcium release 

**Figure 1:**
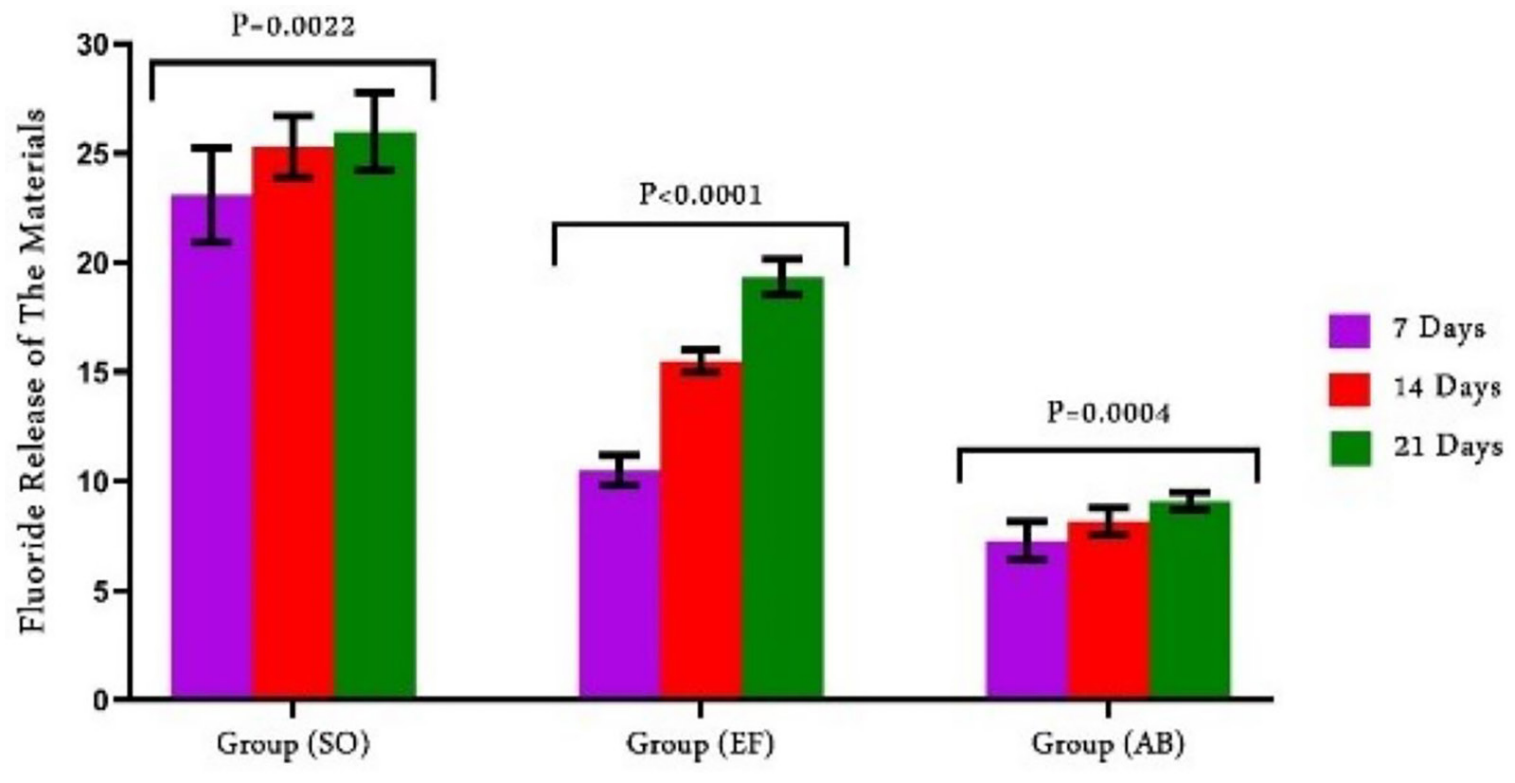
Comparison of fluoride release from the restorative materials.

Figure 1 demonstrate a comparative assessment of fluoride release regarding the time and restorative materials. There are statistically significant differences between all groups (*P*<0.0001). SO group recorded the highest value of fluoride ions (23.08±2.176), followed by EF (10.49±0.6740), while AB recorded the lowest ones (7.280±0.8766). After seven days, fluoride concentrations significantly increased in all groups. After 14 days, EF and AB showed a large rise, but SO showed no discernible change.

**Figure 2:**
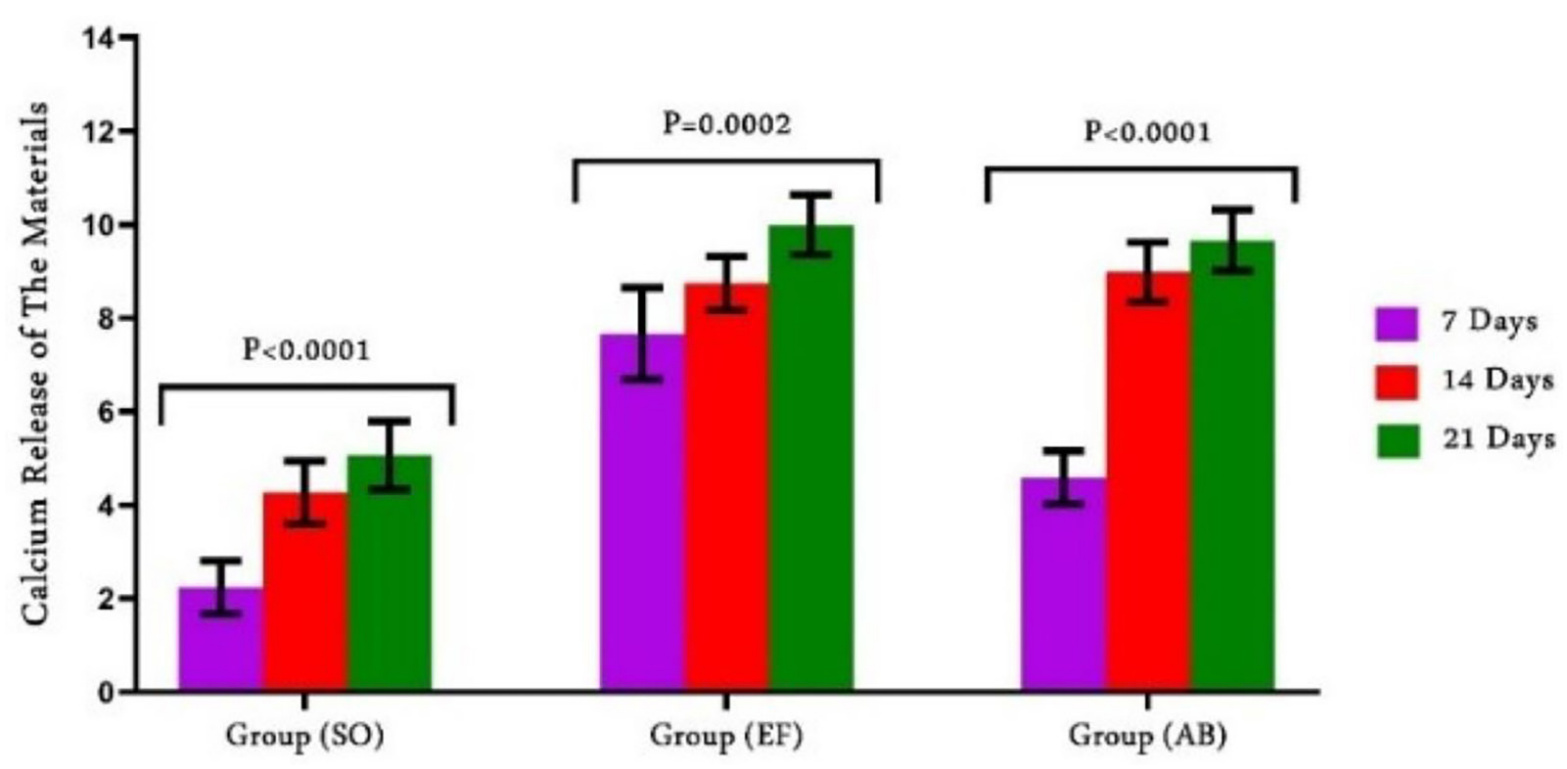
Comparison of calcium release from restorative the materials.

Figure 2 show a comparative assessment of calcium release regarding the time and restorative materials. A statistically significant difference among all groups (*P*<0.0001) was recorded. At every interval, SO recorded the lowest value (2.240±0.570), while EF (7.660±0.9789) and (4.590±0.5724) released the highest calcium ions. After 14 days, the calcium concentrations in EF and SO significantly increased. After 14 days, AB showed no discernible change.

2. Micro-hardness 

**Figure 3:**
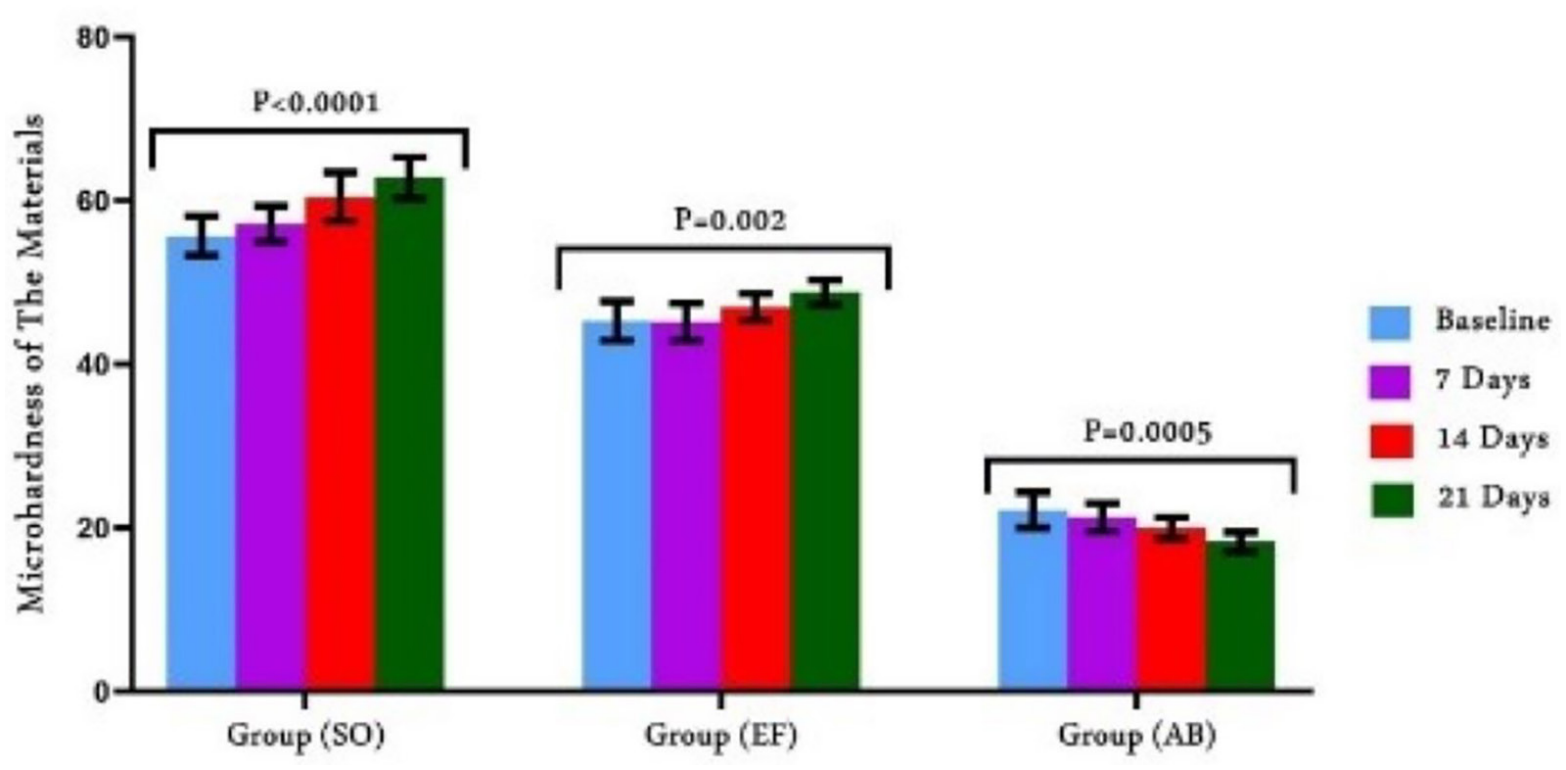
Comparison of surface hardness of the restorative materials.

Figure 3 display the results of VHN for each group based on storage intervals. Materials and storage durations had a statistically significant interaction (*P* ˂ 0.05). Comparison of the values among the investigated materials at baseline (1 h dry storage) recorded statistically significant differences (*P*<0.0001). The aging phase significantly affected VHN. SO recorded the highest values (55.70±2.406) of VHN both earlier and later storage procedure, followed by EF (45.30±2.406) and AB (22.20±2.201) which had the lowest VHN. After water storage, AB exhibited a substantial drop in VHV (*p*<0.0005), while SO and EF showed a significant rise in VHN relative to baseline data.

3. Enamel Demineralization Resistance evaluation 

Microhrdness of the demineralized enamel was significantly inferior to the baseline one for all materials (*p* < 0.0001) as shown in  Table 2. Significant differences of microhardness values of demineralized enamel filled with the different materials were noticed (*P*<0.0001). Demineralized enamel filled with SO recorded the lowest value (219.4±8.299) while EF recorded the highest one (251.8±8.669) followed by AB (239.6±9.773).

4. Elemental Analysis using Energy Dispersive XRay (EDX) 

**Figure 4:**
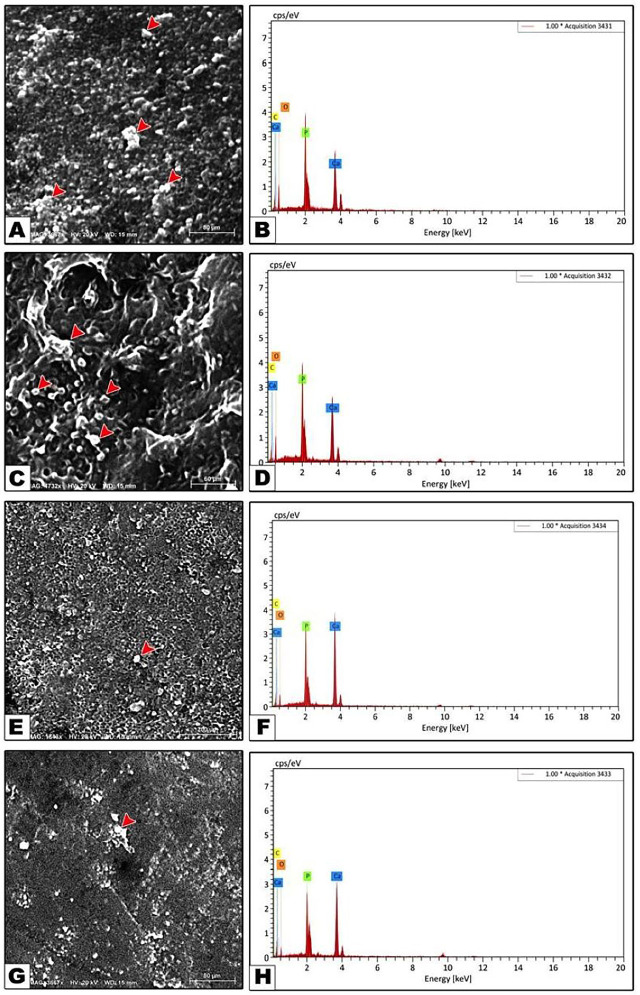
Scanning electron micrographs of demineralized enamel surface filled with the restorative materials and the elemental analysis results: Control group: A&B, SO group: C&D, AB group: E&F. and EF group: G&H.

The percentages of calcium and phosphorus concentration on the enamel adjacent to restorative margins (10µm) are shown in Figure 4. Demineralization dramatically changed the levels of Ca and P, according to the study’s findings. However, Ca levels considerably increased following restoration with EF and AB, but there were no discernible changes in Ca levels following restoration with SO when compared to the control group. Following restoration, *P* levels significantly increased for all studied restorative materials, with AB showing the highest values.

5. Micromorphological Analysis Using Scanning Electron Microscope (SEM) 

 In addition to the positive control group without filling, Figure 4 displays representative SEM/EDX pictures of the calcium and phosphorous weight percentage of the demineralized enamel surface filled with various filling materials, SO, EF, and AB. Due to enamel demineralization, the control group’s enamel surface displayed severe cracks, significant porosity, and abnormalities. Additionally, areas of the enamel surface that were stripped of enamel were visible, revealing the enamel prisms. Together with the loss of prismatic structure, the enamel’s increased porosities were observed (shown with an arrow). SO displayed the same outcomes as the control group. More reduction in AB’s pores and cracks. EF showed that the enamel rods had melted, decreasing the enamel’s porous structure and a smooth surface was noticed. A comparison of the several groups showed that each group differed significantly from the others, was displayed in Figure 4 Ca and P wt.% were increased with enamel filled with EF and AB recorded while the control group and enamel filled with SO exhibited decreased values.

In  Table 3, the Pearson correlation coefficient is displayed. Fluoride release showed a positive correlation with surface hardness and a negative correlation with enamel demineralization resistance. Regarding calcium release, a positive correlation with enamel demineralization resistance and a negative correlation with surface hardness was noticed.

## Discussion

The present study was conducted to estimate the cumulative fluoride and calcium ion release from newly introduced bioactive materials and evlauted the impact on the surface hardness and enamel demineralization resistance. Distallid water was selected as storage medium due to its convenience of use and capability to deliver precise fluoride release measurements free from the possible influence of organic compounds found in artificial saliva (16). Ion-selective electrodes characterized by an easy and appropriate system for quantifying fluoride release but it is unable to distinguish free fluoride ions from fluoride compound released from materials. Ion chromatography (IC) does not detect fluoride complexes while able to estimate little concentrations of fluoride ions. Since free fluoride ions can increase tooth resistance to secondary caries attacks surrounding the restorations (16), IC was used to measure ion release from the different materials

There were significant differences among F and Ca ion release of the different materials. So, the null hypothesis is rejected. F releasing capacity was listed as SO > EF > AB while Ca releasing capacity was listed as EF > AB > SO at all intervals. The material’s fluoride content and the amount of fluoride emitted were thought to be directly correlated. This fact could account for the variations in fluoride release among the materials used in this study (17). A prior work (18) found that SO had a high fluoride content through the elemental analysis of the material, which revealed that SO contain a high fluoride and low calcium content. SO is considered an advancement of resin modified GIC it contains fluroaluminosilicate and silanized non-reactive fillers of different sizes bonded to resin content. Theoretically, this partially water-based substance encourages ion and water exchange with the oral environment. The composition of the fillers causes the release of calcium, fluoride, and aluminum ions (19).

EF showed higher release of F and Ca ions compared to AB. Both materials exhibited regular ions release that increased from the first week to the third one. Similar results (15,20) supported our findings, Rifai *et al*. (21) compared Activa to EF for ion release and concluded that glass hybrid material performs better than composite materials. Hokii *et al*. (22) related the fluoride release from different restorative materials and established that Activa discharged less fluoride than glass hybrids and glass ionomers. Vicente *et al*.’s investigation (23), in contrast to ours, found that Activa releases a considerable concentrations of fluoride ions. This is explained by the material’s exposure to acidic pH media in their study (pH 3, 5), which caused a notable rise in the ion release rate during a specific measurement time. Instead of using a composite made of polymers, EF is basically a glass ionomer cement that is depend on an acid-base reaction from a salt component. The hydrogel matrix phase, which results from the acid-base reaction, forms in a very thick layer following water absorption and may be the cause of the fluoride release (24). Stronium ions added to the EF forming SrF2 which is divided into easier components releasing more F ions (25). The fluoride in AB is derived from sodium fluoride, which in some studies was reactive glass filler (21.8 wt.%) and in others, bioactive glass (BAG) (55.4 wt.%), conditions the release of further ions, including calcium and phosphorus, in addition to fluoride, preventing demineralization. According to Bueno *et al*., (26) materials didn’t discharge fluorides and other ions in the same manner since fluoride is readily trapped inside the matrix. These variations are mostly explained by the composition and the nature of the setting reaction of the restorative material.

Significant variations in MH values were found related to the time and material. So the second null hypothesis was disproved. At various intervals, SO had the greatest MH values, followed by EF, while AB had the lowest values. Because SO has a high load of reactive glass filler, which is round and small, the material has a high abrasion resistance (27). This is in line with the results of earlier research that found a positive association between the hardness and both filler volume and size, so higher hardness values were seen as the filler load increased (28). Compared to AB, EF recorded higher hardness values. Valanezhad *et al*. (29) found that when the number of bioactive glass particles increases, so do the physical properties of the materials. Regarding the structure of EF, novel ultra-fine, extremely reactive glass hybrid fillers are integrated. When paired with a greater molecular weight polyacrylic acid, this novel hybrid glass formulation increases ion availability, enhances matrix construction, and produces a noticeably stronge matrix assembly (7). Filler loading in AB is low (56%) which is frequently bioactive glass fllers so reduction of surface hardness was reported in previous studies (30).

Bidirectional alterations of MH were observed with aging of the restorative materials. This behavior happened as a result of two processes that had conflicting effects on the material’s micromechanical characteristics and led to MH changes: (I) Setting reaction is accomplished in a slow manner that may finished for about one month in composites and glass hybrid cement (31). and (II) water absorption from the surrounding solution, which results in the slow deterioration of material structure (32). Results of our study exhibited an increase in MH values for SO and EF after storage in distilled water for all intervals when compared to the baseline hardness. While AB exhibited decreased values after water storage. The results are in align with the findings of Alzahrani *et al*. (27) who found that the crosslinking reaction of SO is continuous and obstruct the plasticizing influence of the absorbed water. The findings also suggest that a specific amount of time is needed for these materials to reach their maximal level of polymerization. Resin polymerization begins with light curing, while the acid-base reaction advances gradually till additional maturation and eventual maximum hardness. Veček *et al*. (33) found increase of MH amounted to 24% of the initial value for EF. The findings are explained by the fact that glass ionomers go through a maturation procedure that is the continuance of the setting reaction rather than being softened by submersion in water, which progressively enhances the material’s mechanical qualities and water is necessary for a continual chemical reaction (14,34). Also, the glass hybrid filler structure of EF is the reason behind EF’s high hardness value. In contrast to our study, Garoushi (35) found that absorbing water then releasing certain ions like fluoride, phosphate and strontium from the glass ionomer matrix stored in an aqueous atmosphere can soften its superficial layers. reducing surface micro-hardness.

Activa displayed a decrease in MH after storage in water, that might be related to the bioactive glass fillers’ solubility and leaching out of the matrix (36). According to a study (37) that assessed various ion-releasing polymers, including AB, found that the mechanical qualities decreased with prolonged storage. This is explained by the chemical composition of AB as it combines diurethane (UDMA) with other methacrylates. This result is consistent with Sideridou *et al*. (38) who discovered that during thermocycling, the UDMA-based material’s microhardness and stability characteristics dramatically dropped. Increased resin matrix causes the material to soften due to increased water sorption, which lowers the surface hardness.

A demineralizing solution was used to construct an artificial demineralized lesion. Factors, such as the rate of saliva flow and dynamic pH fluctuations in the oral cavity result in difficult full reproduction of oral conditions (39). Because enamel substrate has a small microstructure and a non-consistent surface that is liable to flaw, the VHN test is a recognized method for measuring surface microhardness. This process is quick, non-destructive, and relatively simple. Studies that quantitatively calculated the enamel demineralization have also used this technique (40). When compared to the value prior to demineralization, the microhardness of enamel filled with various materials showed a noTable decrease after demineralization. By breaking down the proteins encircling the enamel rods and crystallites, the acids of the demineralizing solution affect the intraprismatic and interprismatic sections. As a result, a mineral component that is present in enamel protein is also eliminated, lowering the amount of calcium and phosphorus in certain regions, resulting in microstructural damage and potential microhardness modifications (41). There are noTable variations in the microhardness values of demineralized enamel filled with various materials. The highest values were reported by demineralized enamel filled with EF, and the lowest by enamel filled with SO.

In addition to VHN values, the demineralization was evaluated by presence of porosities and cracks on the enamel surface and alterations of the calcium and phosphorus levels. The variations in mineral content were observed using SEM/EDS analysis. EDX determine the amount of minerals concentrations in the substrate, while SEM is effective for observing alterations in surface structure and appearance (39). Unfilled and SO-filled enamels recorded irregular porous cracked surface, according to SEM examination. More reduction in pores and cracks for AB. While EF showed a smooth surface free from porosities and cracks.

Ca and P levels were considerably changed by the demineralization process. Compared to the control group, restoration with EF and AB showed a notable rise in Ca levels, but restoration using SO showed no significant changes in Ca levels. Our study’s findings demonstrated how ion-release affects the prevention of adjacent enamel demineralization. Consequently, the null hypothesis was rejected. Microhardness testing and SEM\EDX revealed that the material that performed the best in inhibition of demineralization at the enamel margin is EF followed by AB. This finding is likely due to high level of calcium ions released from EF and AB compared to SO. As shown in  Table 3, Calcium release and enamel demineralization resistance showed a positive correlation, whereas fluoride release showed a negative correlation. This suggests that high calcium release materials demonstrated strong resistance to enamel demineralization. Utilizing a calcium-based product could successfully obstruct the demineralization process because it has been previously established that calcium is released prior to phosphate ions during the demineralization process and that the amount of Ca release is correlated with increased alkalinity (42). Albelasy *et al*. (19) found increased demineralization-inhibiting action for Cention N ( CN) than that of SO both in depth and degree. This was explained by the quick neutralization capacity of CN as result of the high calcium release more than SO despite of high fluoride release of SO (18). Calcium and phosphate ions are harmful to bacteria due to increased alkalinity and neutralizeation of intraoral pH. So release of these ions is thought to be the cause of the bioactive glass’s antibacterial action.

According to Savarino *et al*. (43), following immersion in demineralizing cariogenic solution, fluoride-releasing materials were incapable to inhibit the process of demineralization at the enamel margin near the restoration. May *et al*. (44) found that fluoride is released by restorative materials have not been demonstrated to prevent tooth demineralization. The results are not consistent with Donly *et al*. (45), who found that the quantity of demineralization inhibition correlated with fluoride release from RMGI materials.

## Conclusions

Along with the outcomes of the current study, it can be determined that surface hardness and enamel demineralization resistance are related to calcium release from the different bioactive restorative materials. These properties have different values depending on the composition of the material. Glass hybrid materials release more calcium ions than composite materials and recorded high enamel demineralization resistance with adequate surface hardness qualities.

## Figures and Tables

**Table 1:** Used materials in the study.

Product	Composition	Manufacturer
Surefil one Self-Adhesive Composite Hybrid	Highly dispersed silicon dioxide, water, , polycarboxylic acid (MOPOS), acrylic acid, ytterbium fluoride, self-cure initiator, bifunctional acrylate (BADEP), pigments, camphorquinone, and aluminum-phosphor-strontium-sodium fluoro-silicate glass	Dentsply Sirona, Konstanz, Germany
Equia forte HT fil Bulk-fill glass hybrid restorative system	Powder: fluoroaluminosilicate glass. Water and polybasic carboxylic acid are liquids.	GC, Tokyo, Japan
Activa-Bioactive restorative	Diurethane and methacrylates are combined with water, patented rubberized resin (Embrace), 21.8 % of glass fillers, inorganic filler (56 %), and modified polyacrylic acid (44.6 %).	Pulpdent, Corporation, Watertown, MA, USA

**Table 2:** Comparison of Microhardness values of enamel restored with restorative material.

	Baseline	Demineralized	P-Value (Paired T-test)
SO	273.4±7.287	219.4±8.299	<0.0001
EF	274.1±11.11	251.8±8.669^a^	<0.0001
AB	275.0±11.66	239.6±9.773^ab^	<0.0001
P-Value	0.89	<0.0001	

*P* value < 0.05 is considered significant. a: significant with Group (SO), b: significant with Group (EF).

**Table 3:** Pearson correlation coefficient (r) between ion release, surface hardness, and enamel demineralization resistance of the restorative materials.

Correlation between variables	Pearson coefficient (r)	P- value
Fluoride release x Surface hardness	0.9093	<0.0001
Fluoride release x enamel demineralization resistance	-0.4988	0.0050
Calcium release x Surface hardness	-0.4578	<0.0001
calcium release x enamel demineralization resistance	0.7769	<0.0001

## Data Availability

The datasets used and/or analyzed during the current study are available from the corresponding author.

## References

[1] Mitwalli H, AlSahafi R, Alhussein A, Oates TW, Melo MAS, Xu HHK (2022). Novel rechargeable calcium fluoride dental nanocomposites. Dent Mater J.

[2] Kim MJ, Lee MJ, Kim KM, Yang SY, Seo JY, Choi SH (2021). Enamel demineralization resistance and remineralization by various fluoride-releasing dental restorative materials. Materials.

[3] Heintze SD, Loguercio AD, Hanzen TA, Reis A, Rousson V (2022). Clinical efficacy of resin-based direct posterior restorations and glass-ionomer restorations - An updated meta-analysis of clinical outcome parameters. Dent Mater.

[4] Simila HO, Boccaccini AR (2022). Sol-gel bioactive glass containing biomaterials for restorative dentistry: a review. Dent Mater.

[5] Ilie N (2022). Resin-based bulk-fill composites: tried and tested, new trends, and evaluation compared to human dentin. Materials.

[6] Klee JE, Renn C, Elsner O (2020). Development of novel polymer technology for a new class of restorative dental materials. JAdhes Dent.

[7] Šalinović I, Stunja M, Schauperl Z, Verzak Ž, Malčić AI (2019). Rajić VB. Mechanical Properties of High Viscosity Glass Ionomer and Glass Hybrid Restorative Materials. Acta Stomatol. Croat.

[8] Croll TP, Berg JH, Donly KJ (2015). Dental repair material: a resin modified glass-ionomer bioactive ionic resin-based composite. Compend Contin Educ Dent.

[9] Lassila L, Garoushi S, Vallittu PK, Säilynoja E (2016). Mechanical properties of fiber reinforced restorative composite with two distinguished fiber length distribution. J Mech Behav Biomed Mater.

[10] Ilie N, Stawarczyk B (2016). Evaluation of modern bioactive restoratives for bulk-fill placement. J Dent.

[11] Moreau JL, Xu HHK (2010). Fluoride releasing restorative materials: effects of pH on mechanical properties and ion release. Dent Mater.

[12] Alshabib A, Silikas N, Algamaiah H, Alayad AS, Alawaji R, Almogbel S (2023). Effect of Fibres on Physico-Mechanical Properties of Bulk-Fill Resin Composites. Polymers (Basel).

[13] Filemban H, Bhadila G, Wang X, Melo MAS, Oates TW, Hack GD (2022). Effects of thermal cycling on mechanical and antibacterial durability of bioactive low-shrinkage-stress nanocomposite. J Dent.

[14] Karakaş SN, Küden C (2022). AFM and SEM/EDS characterization of surfaces of fluorine-releasing bulk-fill restorative materials aged in common liquids. J Oral Sci.

[15] Ruengrungsom C, Burrow MF, Parashos P, Palamara JE (2020). Evaluation of F, Ca, and P release and microhardness of eleven ion-leaching restorative materials and the recharge efficacy using a new Ca/P containing fluoride varnish. J Dent.

[16] Okte Z, Bayrak S, Fidanci UR, Sel T (2012). Fluoride and aluminum release from restorative materials using ion chromatography. J Appl Oral Sci.

[17] Davis HB, Gwinner F, Mitchell JC, Ferracane JL (2014). Ion release from, and fluoride recharge of a composite with a fluoride-containing bioactive glass. Dent Mater.

[18] Abouelleil H, Attik N, Chiriac R, Toche F, Ory A, Zayakh A (2024). Comparative study of two bioactive dental materials. Dent Mater.

[19] Albelasy EH, Chen R, Fok A, Montasser M, Hamama HH, Mahmoud SH (2023). Inhibition of Caries around Restoration by Ion-Releasing Restorative Materials: An In Vitro Optical Coherence Tomography and Micro-Computed Tomography Evaluation. Materials (Basel).

[20] Di Lauro A, Di Duca F, Montuori P, Dal Piva AMO, Tribst JPM, Borges ALS (2023). Fluoride and Calcium Release from Alkasite and Glass Ionomer Restorative Dental Materials: In Vitro Study. J Funct Biomater.

[21] Rifai H, Qasim S, Mahdi S, Lambert MJ, Zarazir R, Amenta F (202). In-vitro evaluation of the shear bond strength and fluoride release of a new bioactive dental composite material. J Clin Exp Dent.

[22] Banic Vidal LS, Veček NN, Šalinović I, Miletić I, Klarić E, Jukić Krmek S (2023). Short-Term Fluoride Release from Ion- Releasing Dental Materials. Acta Stomatol Croat.

[23] Hassan R, Aslam Khan MU, Abdullah AM, Abd Razak SI (2022). A Review on Current Trends of Polymers in Orthodontics: BPA-Free and Smart Materials. Polymers (Basel).

[24] Al-Eesa NA, Wong FSL, Johal A, Hill RG (2017). Fluoride containing bioactive glass composite for orthodontic adhesives - ion release properties. Dent Mater.

[25] Brzović-Rajić V, Miletić I, Gurgan S, Peroš K, Verzak Ž, Ivanišević-Malčić A (2018). Fluoride Release from Glass Ionomer with Nano Filled Coat and Varnish. Acta Stomatol Croat.

[26] Bueno LS, Silva RM, Magalhães APR, Navarro MFL, Pascotto RC, Buzalaf MAR (2019). Positive correlation between fluoride release and acid erosion of restorative glass-ionomer cements. Dent Mater.

[27] Alzahrani B, Alshabib A, Awliya W (2023;19). Surface hardness and flexural strength of dual-cured bulk-fill restorative materials after solvent storage. BMC Oral Health.

[28] Shah PK, Stansbury JW (2014). Role of fller and functional group conversion in the evolution of properties in polymeric dental restoratives. Dent Mater.

[29] Valanezhad A, Odatsu T, Udoh K, Shiraishi T, Sawase T, Watanabe I (2016). Modification of resin modified glass ionomer cement by addition of bioactive glass nanoparticles. J Mater Sci Mater Med.

[30] Francois P, Fouquet V, Attal JP, Dursun E (2020). Commercially Available Fluoride-Releasing Restorative Materials: A Review and a Proposal for Classification. Materials (Basel).

[31] Par M, Lapas-Barisic M, Gamulin O, Panduric V, Spanovic N, Tarle Z (2016). Long Term Degree of Conversion of two Bulk-Fill Composites. Acta Stomatol Croat.

[32] Szczesio-Wlodarczyk A, Sokolowski J, Kleczewska J, Bociong K (2020). Ageing of Dental Composites Based on Methacrylate Resins-A Critical Review of the Causes and Method of Assessment. Polymers (Basel).

[33] Veček NN, Par M, Sever EK, Miletić I, Krmek SJ (2022). The Effect of a Green Smoothie on Microhardness, Profile Roughness and Color Change of Dental Restorative Materials. Polymers (Basel).

[34] Nicholson JW (2018). Maturation processes in glass-ionomer dental cements. Acta Biomater Odontol Scand.

[35] Garoushi S, Vallittu PK, Lassila L (2018). Characterization of fluoride releasing restorative dental materials. Dent Mater J.

[36] Yun J, Burrow MF, Matinlinna JP, Wang Y, Tsoi JKH (2022). A Narrative Review of Bioactive Glass-Loaded Dental Resin Composites. J Funct Biomater.

[37] Daabash R, Alshabib A, Alqahtani MQ, Price RB, Silikas N, Alshaaf MM (2022). Ion releasing direct restorative materials: key mechanical properties and wear. Dent Mater.

[38] Sideridou ID, Karabela MM, Bikiaris DN (2007). Aging studies of light cured dimethacrylate-based dental resins and a resin composite in water or ethanol/water. Dent Mater.

[39] Šalinović I, Schwendicke F, Askar H, Yassine J, Miletić I (2023). Effects of Ion-Releasing Materials on Dentine: Analysis of Microhardness, Appearance, and Chemical Composition. Materials (Basel).

[40] Shah SA, Sharma M, Ismail PMS, Babaji P, Mohammed A, Malik B (2024). Evaluation of Remineralizing Capacity of Tricalcium Phosphate, Nano-Hydroxyapatite and Ozone Remineralizing Agents on the Artificial Carious Lesion. Indian J Dent Res.

[41] Kamath U, Sheth H, Mullur D, Soubhagya M (2013). The effect of Remin Pro® on bleached enamel hardness: an in-vitro study. Indian J Dent Res.

[42] Buzalaf MA, Hannas AR, Kato MT (2012). Saliva and dental erosion. J Appl Oral Sci.

[43] Savarino L, Saponara Teutonico A, Tarabusi C, Breschi L, Prati C (2002). Enamel microhardness after in vitro demineralization and role of different restorative materials. J Biomater Sci Polymer Edn.

[44] May E, Donly KJ (2017). Fluoride release and re-release from a bioactive restorative material. Am J Dent.

[45] Donly KJ, Liu JA (2018). Dentin and enamel demineralization inhibition at restoration margins of Vitremer, Z 100 and Cention N. Am J Dent.

